# Xanthine Oxidase—A Personal History

**DOI:** 10.3390/molecules28041921

**Published:** 2023-02-17

**Authors:** Russ Hille

**Affiliations:** Department of Biochemistry, University of California, Riverside, CA 92521, USA; russ.hille@ucr.edu

**Keywords:** xanthine oxidase, molybdenum enzyme, electron transfer, electron paramagnetic resonance spectroscopy

## Abstract

A personal perspective is provided regarding the work in several laboratories, including the author’s, that has established the reaction mechanism of xanthine oxidase and related enzymes.

## 1. In the Beginning…

**Figure 1:**
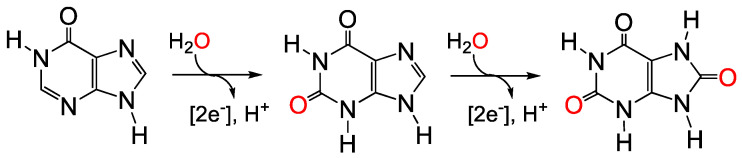
The reactions catalyzed by xanthine oxidase. Hypoxanthine is converted to xanthine, then on to uric acid. The oxygen atom incorporated is derived from water.

Xanthine oxidase catalyzes the oxidative hydroxylation of hypoxanthine to xanthine and xanthine to uric acid, the final two steps in purine catabolism in humans, as shown in Figure 1. It is extremely broadly distributed in nature as all organisms must metabolize purines. The enzyme is a dehydrogenase in most organisms, passing the reducing equivalents it obtains from substrate hydroxylation to NAD+. The enzyme from mammalian sources, although initially expressed as a dehydrogenase, is typically isolated as an oxidase, passing the reducing equivalents to O_2_ rather than NAD+. For this reason, the enzyme is often referred to as xanthine oxidoreductase. The reaction is unusual if not unique among those enzymes catalyzing the hydroxylation of carbon centers in that it utilizes an oxygen atom derived from water rather than O_2_, and generates rather than consumes reducing equivalents. This activity was likely critical to the earliest organisms evolving in the anaerobic environment of the early Earth, and indeed genomics studies have traced its origins back to the Last Universal Common Ancestor (LUCA) to all extant organisms [1]. The enzyme from cow’s milk was one of the first enzymes to be purified to homogeneity, as reported by Dixon and Thurlow in 1924 [2,3]. It would be another 30 years before Richert and Westerfeld showed that the enzyme possessed molybdenum [4] and not until 1966 that Bray and Meriwether demonstrated unequivocally that certain EPR signals manifested by the enzyme were due to molybdenum, which became reduced in the course of the oxidative hydroxylation of xanthine to uric acid [5]. This work involved the isolation and characterization of enzyme from the milk of a cow that had been injected with ^95^Mo-labeled molybdate (^95^Mo having a nuclear spin of 5/2, yielding sextets of lines in the EPR spectrum)—an impressive isotope substitution experiment involving a (very large) eukaryote. 

I was first introduced to xanthine oxidase in graduate school at Rice University, ca. 1975. I was working in the laboratory of John S. Olson at the time, attempting to develop methods to distinguish ligand binding to the α and β subunits of hemoglobin, first by inserting spectroscopically distinct heme derivatives into one or the other subunit of the protein and later (and much more successfully) using NO, the α and β subunits of nitrosylhemoglobin giving distinct EPR signals, with that of the α subunits differing in the R and T states [6,7]. I had two good friends, first Mike Davis and later Arturo Porras, who at the time were pursuing their PhDs next door in Graham Palmer’s laboratory and were working on xanthine oxidase. I remember early on being impressed at the complexity of the enzyme, with its molybdenum center, two different Fe_2_S_2_ iron-sulfur clusters and FAD, as well as by the UV/visible and EPR spectral signatures of these centers. Mike was looking at the reaction of the enzyme with lumazine, the pteridine analog to the purine xanthine, the interest being that while there was no significant UV/visible absorbance attributable to the molybdenum center of the enzyme, two different charge-transfer complexes with long-wavelength absorbance were seen in the course of the reaction with lumazine [8,9]. The first of these was a charge-transfer complex between the oxidized molybdenum center and substrate lumazine, and the second a complex between reduced molybdenum and the product violapterin. It was interesting that two such reaction intermediates in the course of substrate hydroxylation would appear when the molybdenum center itself seemed devoid of absorbance.

**Figure 2:**
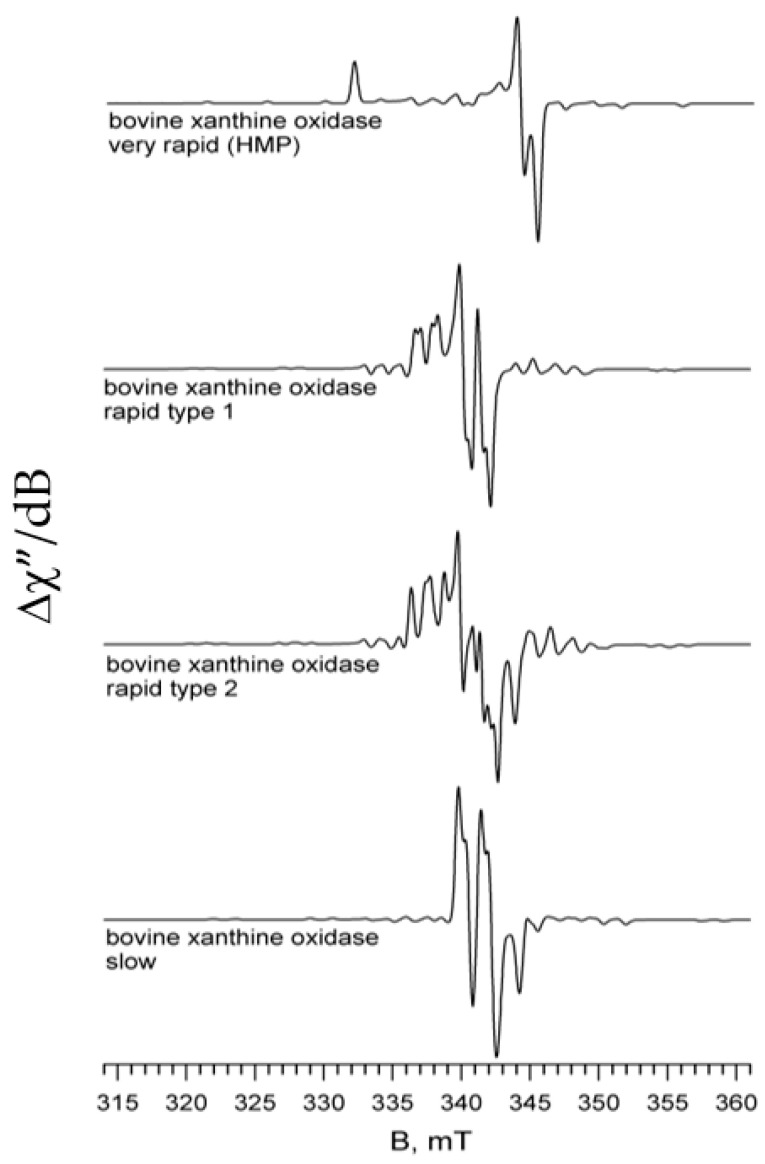
Mo(V) EPR signals exhibited by xanthine oxidase. From top to bottom: “Very Rapid”, “Rapid Type 1”, Rapid Type 2” and “Slow” signals.

Palmer had moved to Rice after a number of years at the University of Michigan, where he had studied xanthine oxidase in collaboration with Vincent Massey. Palmer had in fact been Massey’s first graduate student at the University of Sheffield, and as a post-doctoral scholar in Helmut Beinert’s laboratory at the University of Wisconsin had, among other projects, worked on xanthine oxidase with a visiting scientist from the Chester Beatty Research Institute in London, Robert C. Bray. Having demonstrated that xanthine oxidase contained molybdenum, and that the metal became reduced in the course of the reaction with xanthine, Bray was using a freeze-quench apparatus of his own design to follow the kinetics of substrate hydroxylation by EPR. The work in Beinert’s laboratory demonstrated that several Mo(V) EPR signals arose and decayed in the course of the reaction [10,11]. These eventually came to be known as “Very Rapid”, “Rapid” and “Slow”, based on the time scales on which they appeared and decayed, and the first two signals were immediately recognized as being catalytically relevant. These are shown in Figure 2. Palmer brought the xanthine oxidase project with him to the University of Michigan, and began a long and productive collaboration with Massey.

Olson had himself worked with Palmer and Massey on xanthine oxidase as a post-doctoral scholar at the University of Michigan. He had carried out his graduate work on hemoglobin with Quentin Gibson at Cornell University (famously, he published a research paper from each of the several laboratory rotations he did in his first year in the course of selecting an advisor). Upon graduation, Olson took a post-doctoral position with Massey (who had been a colleague of Gibson’s at the University of Sheffield) and worked on xanthine oxidase in collaboration with Palmer and his student at the time, David Ballou. Olson had applied for an NIH post-doctoral fellowship for support during his work at Michigan, and George Schroepfer, the Chair of the new Department of Biochemistry at Rice, had served on the review committee. He was so impressed with Olson’s application that he invited Olson to apply for a faculty position at Rice while Olson was still finishing up at Cornell, and after a successful interview an offer was extended. It thus transpired that Olson arrived for his post-doc in Ann Arbor having already accepted a faculty position at Rice. (Unbeknownst to him at the time, and quite ironically, Palmer had also been recruited to a senior position in the Biochemistry Department at Rice.) Olson spent only nine months with Massey, but the resulting two papers that appeared back-to-back in *J. Biol. Chem.* totaled an unheard-of 40 pages [12,13]. The first of these dealt with the oxidative half-reaction of the catalytic cycle, the reaction of reduced enzyme with O_2_, and demonstrated that the reaction was distinctly biphasic with the first five reducing equivalents removed from the fully (six-electron) reduced enzyme in the fast phase of the reaction and the sixth electron removed in the slow phase of the reaction. At least two equivalents of superoxide, O_2_•^−^, were thus expected to form, one during the fast phase and the second in the slow phase of reoxidation. The second of the two papers, immodestly entitled “The Reaction Mechanism of Xanthine Oxidase”, was indeed a breakthrough in the field and over the years has profoundly shaped my own thinking about the enzyme. In it, the behavior of the enzyme was described in the context of a rapid equilibrium model in which reducing equivalents introduced in pairs in the course of substrate oxidation equilibrated very rapidly (i.e., much faster than the rates at which they were introduced into the enzyme) among the four redox-active centers and distributed themselves in the intermediate two- and four-electron reduced enzyme species in the reaction (a total of six electrons being required to fully reduce the enzyme) simply according to the reduction potentials of the several centers. Although the calculations were rather tedious given the number of potentials involved, the model was conceptually straightforward and accounted for a number of properties of the enzyme that had defied explanation up to that point. Importantly, it predicted the relative reduction potentials of the several centers in the enzyme, potentials that were ultimately confirmed in potentiometric work later carried out by my friend Arturo Porras in Palmer’s laboratory at Rice [14].

On the basis of the above, it might have seemed inevitable that I would work on xanthine oxidase with Vincent Massey after completing my Ph.D. I had in fact developed an interest in oxidation-reduction enzymology and the type of rapid-reaction kinetic applications in particular in which Massey excelled, and in the eventuality, I did approach him about a post-doctoral position. I had suggested, however, working on the simple flavoprotein glycollate oxidase rather than xanthine oxidase since the former enzyme had been the subject of a mock grant proposal I had had to prepare and defend as part of the Ph.D. program in Biochemistry at Rice. With letters of recommendation from Palmer and Olson, I was indeed offered a post-doctoral position, but Massey encouraged me to work on xanthine oxidase instead of glycollate oxidase. He had a specific project in mind, substituting the natural FAD of the enzyme with chemically modified flavins of varying reduction potential to see if the properties of the enzyme changed in ways predicted by the rapid equilibrium model. It sounded very interesting. The deal was sealed when I won an appointment from the Michigan Society of Fellows, and I left for Ann Arbor.

## 2. Michigan Days

**Figure 3:**
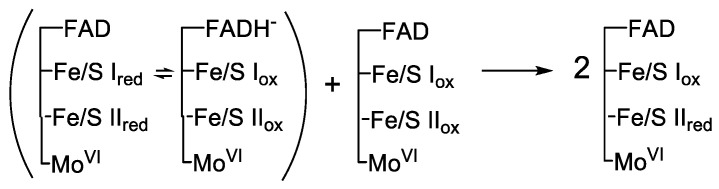
The Comproportionation of XO_2e_− with XO_ox_. Intermolecular electron transfer from XO_2e_−, containing approximately equal amounts of the two electron distributions shown in parentheses, with XO_ox_ yields two molecules of XO_1e_−, which has the single electron predominantly in the higher-potential Fe/S II center. The molybdenum center remains largely oxidized in both XO_2e_− and XO_1e_−.

I drove with my brother from Houston to Ann Arbor through solid rain in the wake of Hurricane Debra in early September, 1978. It was a miserable three-day slog, but the weather improved just as we arrived in Ann Arbor one afternoon and clear skies broke. It seemed prophetic. Ann Arbor is one of those towns, like Bowling Green or Miami of Ohio, that you heard of regularly on the old Prudential College Scoreboard television program, and these places had always held an almost mythical status in my mind because of their association with college football. When I arrived in the Fall of 1978, Ann Arbor’s population was about 80,00… except on Saturdays in the Fall when the University of Michigan football team had a home game, in which case the population doubled. Quite unlike Houston, Ann Arbor had (and still has) a spectacular autumn season. I settled in at the Massey lab and had in short order completed my first of many preparations of xanthine oxidase. By way of familiarizing myself with the system, I decided to repeat many of the experiments that Olson had reported in those forty pages of *J. Biol. Chem*. One in particular gave me fits. It involved reacting the enzyme with a substoichiometric amount of xanthine under anaerobic conditions and following the evolution of the difference spectrum relative to oxidized enzyme with time. According to the rapid equilibrium model, the small amount of two-electron reduced enzyme initially generated would have an approximately equal distribution of the electrons between the fully reduced flavin on the one hand and the two reduced iron-sulfur centers on the other, since one of the iron-sulfur cluster had a high potential and the other a low potential and their average was approximately equal to the midpoint potential of the FAD. Over an hour or two, however, the two-electron reduced enzyme would comproportionate with a molecule of oxidized enzyme to give two equivalents of one-electron reduced enzyme, with the electron residing predominantly on the higher-potential iron-sulfur center, Fe/S II, as shown in Figure 3. The result was the net oxidation of FADH_2_ and reduction of iron- sulfur centers, readily evident in the change in the difference spectrum. The problem was maintaining sufficient anaerobiosis in the specialized anaerobic cuvette used in the experiment, such that there was no detectable reoxidation of the enzyme over the course of an hour or more. I must have repeated the experiment half a dozen times before finally reproducing Olson’s result… after which, like the American experience in Vietnam (still fresh on everyone’s mind at the time), I declared victory and moved on.

The work with the enzyme substituted with chemically modified flavins proved quite successful, and in the eventuality the rapid equilibrium model was borne out: with high-potential flavins such as the 8-chloro derivative, the FAD became reduced much earlier relative to the iron-sulfur clusters in the course of reductive titrations with sodium dithionite than seen with native enzyme, and with low-potential flavin such as the 6-hydroxy derivative, the FAD became reduced much later in the course of the titration [15,16]. These experiments involved following enzyme reduction by UV/visible and EPR, and entailed the use of a specialized titration device for removal of measured aliquots of protein in the course of the titration into an EPR tube; the glassware was quite heavy with three different stopcocks and rested on a fused quartz cuvette that was only 1 mm thick; it was a device that seemed satanically inspired if not designed (the actual culprit turned out to be Graham Palmer). I carried out many day-long experiments with the thing, burning through at least three 4-g enzyme preparations in the process, and miraculously never broke it. In later work, we showed that enzyme with flavins having low reduction potential exhibit low levels of steady-state turnover [16]. The correlation between steady-state turnover activity and flavin reduction potential turned out not to be due to low rates of reaction with O_2_ of the modified flavins but instead to an unfavorable equilibrium distribution of electrons in partially reduced enzyme forms encountered in the course of turnover: to the extent that the distribution favored reduction of molybdenum and iron-sulfur centers, and oxidized flavin, the flavin of partially reduced enzyme forms accumulating in the course of catalysis was simply in the wrong oxidation state to react with O_2_, and similarly, to the extent that the molybdenum was reduced, it was unable to react with xanthine.

## 3. The Oxidative Half-Reaction of Xanthine Oxidase and Intramolecular Electron Transfer

**Figure 4:**
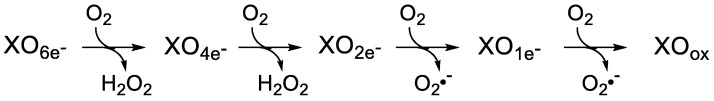
The reaction of reduced xanthine oxidase with O_2_. The first four reducing equivalents are removed in two pairs to generate peroxide. The final two reducing equivalents leave individually to generate two equivalents of superoxide.

With the work on the enzyme with chemically modified flavins successfully completed, I began to think about what next to do with this enzyme that was so easy to come by. Two things came to mind, first pursuing studies of the oxidative half-reaction of the enzyme, the reaction of reduced enzyme with O_2_, and second devising a way to examine intramolecular electron transfer within the enzyme, a central hypothesis of the rapid equilibrium model being that electron transfer among the several redox-active centers of the enzyme was rapid compared to the ~15 s^−1^ k_cat_. With regard to the oxidative half-reaction, I used cytochrome *c* to track superoxide in the course of the reaction, cyt *c* being rapidly and effectively reduced by the O_2_•^−^ formed in the course of enzyme reoxidation [17], yielding a large and easily quantified absorbance change (using an extinction coefficient, interestingly enough, that Massey had determined years earlier [18]). The results clearly showed that there was a lag in superoxide generation and that only the last two reducing equivalents removed from fully reduced enzyme formed O_2_•^−^, both equivalents in two-electron reduced enzyme (generated on reduction with substoichiometric concentrations of xanthine), resulted in formation of superoxide. The clear implication was that the oxidative half-reaction proceeded with formation of two equivalents of peroxide, followed by two equivalents of superoxide, as indicated in Figure 4. As it turns out, Arturo Porras in Graham Palmer’s lab had done similar experiments that included following peroxide formation explicitly using cytochrome *c* peroxidase [19]. His results and mine agreed completely where they overlapped, and otherwise complemented one another nicely—a happy outcome indeed. The two papers appeared side by side in *J. Biol. Chem.* in 1981. At about the same time, Arturo completed a very impressive series of room-temperature potentiometric experiments with xanthine oxidase that demonstrated the several redox-active centers of the enzyme did indeed have the relative reduction potentials predicted by the rapid equilibrium model [20].

**Figure 5:**
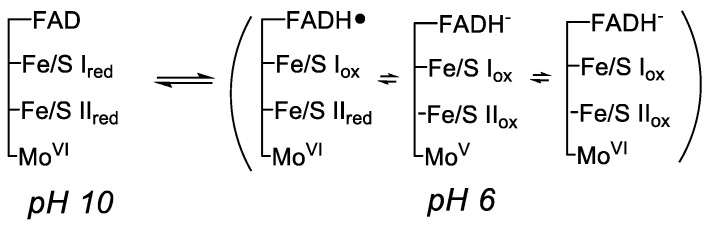
The predominant electron distributions in, as an example, two-electron reduced xanthine oxidase as a function of pH. A pH jump from pH 10 to 6 should result in the net oxidation of Fe/S I and substantial reduction of FAD to either FADH• or FADH^−^.

**Figure 6:**
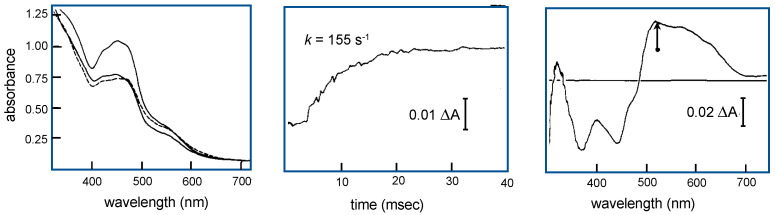
Intramolecular electron transfer within xanthine oxidase as studied by pH jump. **Left**, the absorption spectra of oxidized enzyme and enzyme reduced to the level of approximately 50% at pH 10 (solid line) and 6 (dashed line). **Center**, the kinetic transient seen at 525 nm on jumping the from pH 10 to pH 6. **Right**, the [low pH]-*minus*-[high pH] difference spectrum of partially reduced xanthine oxidase, with the extent of the absorbance change observed kinetically at 525 nm indicated by the arrow; ~50% of the total absorbance change occurs in the ~2-ms dead time of the stopped-flow instrument. After Hille and Massey [21].

To approach the issue of intramolecular electron transfer, I wanted to take advantage of the different pH dependences of the reduction potentials for flavin and iron-sulfur clusters. Being coupled to the uptake of at least one proton, the flavin midpoint potential was expected to be considerably more sensitive to pH than the potentials of the iron-sulfur clusters. At lower pH values, reduction of the flavin in partially reduced enzyme should be preferred, while at higher pH, reduction of the iron-sulfurs should be preferred, as shown in Figure 5. The strategy was to take partially reduced enzyme in dilute buffer at one pH and mix it with anaerobic buffer at another pH under anaerobic conditions and see what happened, as illustrated in Figure 6. In both high-to-low and low-to-high pH jumps, rate constants in the range of 150–300 s^−1^ were observed, with the spectral changes associated with the observed kinetics consistent with electron re-equilibration between flavin and iron-sulfur centers, as expected; the lower-potential iron-sulfur cluster (that designated Fe/S I) was presumed to be principally involved in this equilibration [21]. It turned out that only about half of the expected spectral change at ~525 nm was observed experimentally, indicating that there was a dead time spectral change in the stopped-flow experiment that involved another re-equilibration process. This dead time spectral change was abolished when the molybdenum center was complexed with alloxanthine and rendered redox-inert by locking it in the Mo(IV) state, the conclusion being that the dead time spectral change seen with native protein was due to electron equilibration between the molybdenum (which, like the flavin, was known to take up protons upon reduction) and iron-sulfur centers. In later work, and the only research paper on which I was sole author, I examined the solvent isotope effect on the pH jump kinetics, and found clear evidence that a proton (presumably the N-5 hydrogen of the neutral flavin semiquinone) was in motion as the system traversed the electron-transfer transition state, an early example of proton-coupled electron transfer [22].

The results of the pH jump work were internally consistent, but at odds with studies utilizing flash photolysis to rapidly introduce reducing equivalents into the enzyme and follow subsequent electron equilibration spectrophotometrically [23,24]. There were a number of unresolved technical issues with the flash photolysis technique, but it was clear that alternative experimental approaches would be helpful in resolving the issue. Pulse radiolysis was an attractive alternative to flash photolysis, and so a collaboration was established with Bob Anderson, then at the Gray Laboratory of the Cancer Research Campaign in London, to look at electron transfer within xanthine oxidase using pulse radiolysis [25,26]. There was a considerable amount of time spent on working out the appropriate reaction conditions, but ultimately Bob hit on the idea of using radiolytically generated methylnicotinamide radical, NMN•, to rapidly reduce the enzyme. This worked very effectively in reducing the enzyme, and at the molybdenum center specifically (the reaction again being blocked by complexing the molybdenum center with alloxanthine). After considerable refining of the experimental conditions, we were able to confirm the ~150 s^−1^ rate constant for equilibration of reducing equivalents between the flavin and iron-sulfur clusters seen in the pH jump experiment at low pH, and identify a much faster process with a rate constant of 8500 s^−1^ involving electron equilibration between the molybdenum center and iron-sulfurs (again expected to involve predominantly the lower-potential Fe/S I). The implication was that internal electron transfer within the enzyme proceeded from the molybdenum center, where they were introduced by xanthine, to the iron-sulfur clusters to the FAD, and this was subsequently confirmed by X-ray crystallography (see below).

## 4. A Renewed Focus on the Reductive Half-Reaction: EPR and XAS

By this point, I had become increasingly interested in the mechanism of the reductive half-reaction of xanthine oxidase, the reaction of oxidized enzyme with xanthine that led to its oxidative hydroxylation to uric acid at the enzyme’s molybdenum center. In particular, I was interested in somehow using the same kinds of approaches as employed so successfully by Massey in studying a wide variety of flavoproteins, this despite the fact that the large UV/visible spectral changes seen in the course of the reaction of a flavoprotein were absent in the molybdenum center of xanthine oxidase. Meanwhile, Bob Bray, by now at the University of Sussex, had been continuing to employ EPR to investigate the molybdenum center of xanthine oxidase. Bray had demonstrated that there were two types of “Rapid” signal that differed in the manner of proton coupling and that both possessed bound substrate, as shown in Figure 2 [27,28,29]. He had also shown that the C-8 proton of xanthine was transiently transferred to the molybdenum center in the course of substrate hydroxylation and was strongly coupled to the unpaired electron spin on molybdenum [30]. He provided evidence to suggest that the catalytically essential sulfur that had been thought to be an active site persulfide was instead a Mo=S group that was replaced by a Mo=O group upon reaction with cyanide, and that it was the Mo-SH formed on reduction and protonation that was responsible for the strongly coupled proton seen in the “Rapid” signal [31]. Finally, from quantitative comparison of ^17^O [32,33,34] and ^33^S [35,36] coupling seen in the enzyme with that observed in inorganic model compounds (prepared by, e.g., Tony Wedd at the University of Melbourne, John Enemark at the University of Arizona and Jack Spence at the University of Utah), a structure for the species giving rise to the “Very Rapid” signal was proposed in which product was coordinated to the molybdenum center via the catalytically introduced hydroxyl group, with an unprotonated Mo=S; the “Rapid type 1” signal was proposed to have substrate bound in the active site but not coordinated to the molybdenum, which had both Mo-SH and Mo=O groups. These assignments proved correct in the eventuality, although Bray later abandoned the idea, as discussed further below.

A game-changer in our understanding of the molybdenum center was the development of X-ray absorption spectroscopy, and particularly extended X-ray absorption fine structure (EXAFS) analysis, to study xanthine oxidase and other molybdenum enzymes. From 1979 to 1989, the active site of xanthine oxidase became increasingly clearly defined [37,38,39,40,41,42], with a Mo^VI^OS core (and two or more sulfurs at a longer distance than the Mo=S) in the oxidized enzyme and a Mo^IV^O(SH) in the reduced enzyme; consistent with the earlier EPR work from the Bray laboratory, the Mo=S of oxidized enzyme was replaced by a second Mo=O in the inactive desulfo form of the enzyme. My own contribution to this work was in collaboration with Steve Cramer, then at Exxon Research and Engineering, and in addition to carefully characterizing the oxidized and reduced forms of the enzyme this work involved an investigation of arsenite-inhibited enzyme [39] and complexes of enzyme with violapterin and alloxanthine [40]. The arsenite work demonstrated that the arsenite was coordinated directly to the metal via a sulfur atom (presumably the catalytically labile Mo=S). For the complex of fully reduced enzyme with alloxanthine, the data were consistent with a Mo^IV^O(SH) core with alloxanthine bound via one of its ring nitrogens (presumably occupying the position equivalent to C-8 in xanthine). The results with the violapterin complex were also interpreted as indicating a Mo^IV^O(SH) core, with the product violapterin coordinated to the molybdenum via the catalytically introduced hydroxyl group.

## 5. On My Own

In 1985, about halfway through the pulse radiolysis and EXAFS work described above, I arrived at the Ohio State University to take up an independent faculty position. Massey had been exceptionally generous during the final years of my post-doctoral studies, allowing me to independently pursue the pulse radiolysis and EXAFS collaborations, for example, and he had also agreed to split the National Science Foundation award that had supported me for the previous several years so that I arrived in Columbus with grant support. I only learned much later that my successful renewal of my portion of the NSF award was due in no small part to Massey electing not to submit a renewal of his own, which was extremely gracious of him. (I mentioned this to someone once and they commented he must have been really eager to get rid of me. That may be closer to the truth than I would like to admit!) In any case, one of the first things I was interested in on setting out independently was determining whether there was a catalytically labile oxygen in the molybdenum coordination sphere, a possibility that was being considered in the literature, but which had yet to be demonstrated. Working with Howard Sprecher, a colleague at Ohio State, we worked out a protocol for volatilizing product uric acid with a reagent that quadruply labeled uric acid with trimethylsilyl groups in a way that would not displace the catalytically introduced hydroxyl oxygen at C-8. The enzyme was labeled with 18-O by turnover in H_2_^18^O, then transferred to a H_2_^16^O solution and reacted with substoichiometric xanthine (to ensure no more than one turnover per enzyme molecule). We found the labeled product isolated from the reaction mix to have a mass larger by 2 compared to authentic 16-O labeled uric acid; the converse experiment with unlabeled enzyme in H_2_^18^O yielded unlabeled uric acid [43]. It was clear that there was indeed a catalytically labeled site on the enzyme that was the source of the oxygen atom incorporated into product by the enzyme. We originally considered this to be the Mo=O as this was the only unambiguously identified oxygen ligand to the molybdenum at the time, but in later work using 17-O, we showed that the catalytically labile oxygen of the molybdenum center was strongly and anisotropically coupled to the unpaired electron spin in the “Rapid” EPR signal [44], consistent with it being an equatorial Mo-OH rather than apical Mo=O group, as is now generally accepted.

**Figure 7:**
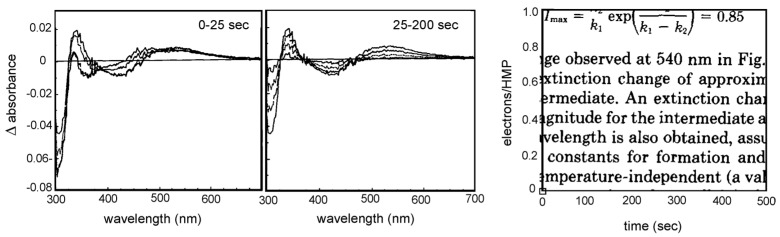
The reaction of xanthine oxidase with 2-hydroxy-6-methylpurine. The evolution of difference spectra (relative to oxidized enzyme) seen on mixing xanthine oxidase with substoichiometric 2-hydroxy-6-methylourine over the first 25 s of reaction (**Left**) and subsequent 200 s (**Center**) under aerobic conditions. A first intermediate with a difference maximum at 470 nm forms in the mixing deadtime and evolves to a second with an absorbance maximum at 540 nm in the first 25 s of the reaction, which subsequently decays over the next 200 s to oxidized enzyme. (**Right**), the time course of formation and decay of the “Very Rapid” EPR signal under the same conditions, demonstrating that the species giving rise to the signal corresponds to the second of these two intermediates. After [46].

With a very talented post-doctoral scholar, Robert McWhirter, we next examined the reaction of enzyme with the slow substrate 2-hydroxy-6-methylpurine, which Bob Bray had previously shown to transiently form very large amounts of the “Very Rapid” EPR signal that was generally believed to be an authentic catalytic intermediate [45]. A key advantage of this substrate was that because the reaction at the molybdenum center was so slow, reducing equivalents did not accumulate in the FAD and iron-sulfur clusters when the reaction was carried out under aerobic conditions. In the eventuality, as illustrated in Figure 7, we were able to identify by UV/visible spectroscopy not one, but two intermediates in the course of substrate hydroxylation at the molybdenum center [46]. The second of these formed and decayed at the same rates as the species, giving rise to the “Very Rapid” EPR signal, a Mo(V) species, and arose from the preceding intermediate on a tens of seconds time scale. Formation of the “Very Rapid” species coincided with the loss of an electron, as determined by cytochrome *c* reduction, indicating that this first intermediate was a Mo(IV) species. Formation of the “Very Rapid” species was thus an oxidative event, meaning that the initial reduction of the molybdenum center was a two-electron event, leading directly from Mo(VI) to Mo(IV). In subsequent pulse radiolysis work, again with Bob Anderson (now at the University of Auckland in New Zealand) we provided explicit evidence that the initial reduction event was indeed a two-electron rather than one-electron process [47]. In other work with 2-hydroxy-6-methylpurine, we showed that partially reducing the enzyme prior to reaction with substrate resulted in the immediate accumulation of the “Rapid Type 1” EPR signal, much more rapidly than the eventual accumulation of the “Very Rapid” species [48]. The ability to reverse the order of appearance of the “Rapid” and “Very Rapid” signals indicated that the “Rapid” species represented a complex of substrate with partially reduced enzyme and did not lie downstream from the species giving rise to the “Very Rapid” signal in a given catalytic cycle. It in fact represented a paramagnetic analog of the Michaelis complex, but one which could not proceed in the catalytic sequence until the molybdenum center was fully reoxidized by electron transfer to the other redox-active centers in the enzyme. In this sense, and counterintuitively, it preceded rather than succeeded the “Very Rapid” species in a given catalytic sequence.

## 6. The Structure of the “Very Rapid” Species

The key question at this point was the manner in which substrate cum product was bound in the molybdenum center of the “Very Rapid” species. With David Britt at the University of California, Davis, we first examined the “Very Rapid” species (generated again using 2-hydroxy-6-methylpurine) by electron spin echo envelope modulation (ESEEM) spectroscopy, and found strong 14-N coupling that was absent in a “Desulfo Inhibited” signal known to not have bound substrate; we interpreted the ^14^N-coupling to noncovalently bound substrate [49]. With a sample prepared in D_2_O, coupling to a deuteron at ~3.2 Å was observed, but no coupling to the methyl group deuterons was observed, indicating they were >4.9 Å from the molybdenum. The results were consistent with the signal-giving species having product coordinated to the molybdenum via the catalytically introduced hydroxyl group at C-8. We later extended this work by examining the ENDOR of 8-^13^C-labeled 2-hydroxy-6-methylpurine (synthesized by a talented student in my lab, Eun-Young Choi), work that was done in collaboration with Brian Hoffman at Northwestern University. Our results [50] were similar to earlier work by David Lowe, Bob Bray and coworkers [51], but whereas they concluded that the Mo-^13^C distance was less than 2.4 Å, consistent with a direct Mo-C bond, we concluded that the distance was 2.7–2.9 Å, consistent with a Mo-O-C linkage. At issue was the extent of sp hybridization at C-8, which influenced the extent of isotropic versus anisotropic coupling in the ^13^C A tensor: sp hybridization yielded a short Mo-C distance of <2.4 Å, with progressively longer distances for sp^2^ (2.65 Å) and sp^3^ hybridization (3.00 Å). Bray and coworkers had assumed (an unrealistic) sp hybridization on the assumption that an sp orbital was more directionally oriented in space than an sp^3^ orbital and therefore more likely to contribute anisotropically to the observed coupling. In fact, an sp-hybridized orbital is considerably more spherical (and less directionally oriented) than a given sp^3^-hybridized orbital and thus yields a shorter estimated Mo-C distance on the basis of the observed anisotropy of the A tensor, rather than a longer one as assumed by Bray and Lowe. This being the case, the data clearly indicated a Mo-C distance too great for direct binding of carbon to the molybdenum. 

**Figure 8:**
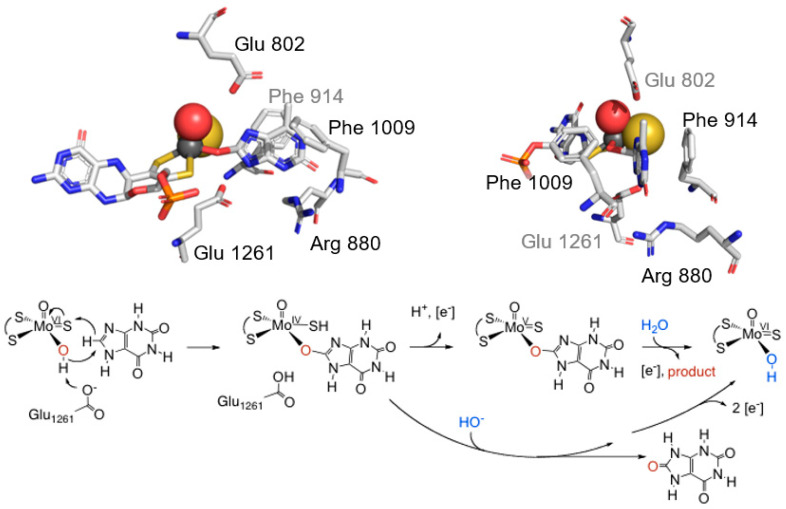
The X-ray crystal structure of xanthine oxidase upon reaction with 2-hydroxy-6-methylpurine. **Upper left**, a view from the side of the active site, showing substrate cum product coordinated to the molybdenum via the catalytically introduced hydroxyl group. **Upper right**, a view looking down the substrate access channel to the active site, rotated approximately 90° about the vertical from the orientation shown at left. The several active site residues discussed in the text are indicated. After Pauff et al., 2008 [52] (PDB B9J). **Bottom**, the reaction mechanism of xanthine oxidase, involving base-assisted nucleophilic attack of the equatorial Mo-OH on C-8 of substrate, followed by hydride transfer to the Mo^VI^=S group to yield Mo^IV^-SH. Breakdown of this intermediate occurs either by product displacement from the molybdenum by solvent hydroxide (and subsequent rapid electron transfer out of the molybdenum center) or electron transfer with product remaining bound to yield the “Very Rapid” species.

The issue of how product was bound in the “Very Rapid” species was ultimately settled using protein crystallography. James Pauff in my laboratory, working closely with my colleague Charles Bell at Ohio State, determined the structure of enzyme complexed with 2-hydroxy-6-methylpurine, which, as shown in Figure 8, clearly showed product coordinated to the molybdenum via the catalytically introduced hydroxyl at C-8, as predicted on the basis of a mechanism in which the equatorial Mo-OH nucleophilically attacked the C-8 carbon of xanthine, with concomitant hydride transfer to the (also equatorial) Mo^VI^=S to yield the two-electron reduced Mo^IV^-SH [52]. The overall structure of the initial catalytic intermediate could thus be formulated as LMo^IV^O(SH)(OR), where L is the enedithiolate-coordinated pyranopterin cofactor and OR the molybdenum-coordinated product. The result was entirely consistent with the reaction mechanism we had previously proposed, as shown in Figure 8. It is to be noted that while a simple arrow-flipping mechanism adequately describes the changes in oxidation state of the participating groups of the active site, Martin Kirk has considered a more accurate description of the molecular orbitals involved and shown that a back-bonding effect between metal and ligands that is fundamentally charge-transfer in nature effectively plays a major role in the charge separation that must necessarily accompany a formal hydride transfer mechanism, thus contributing significantly to transition state stabilization [53].

## 7. X-ray Crystal Structures… At Last!

**Figure 9:**
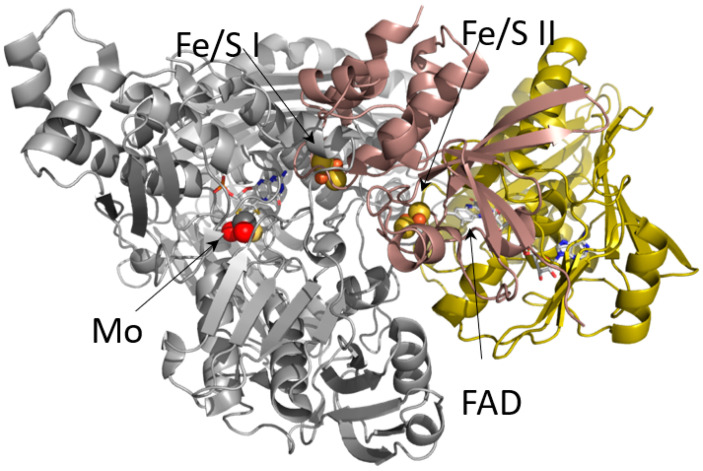
The X-ray crystal structure of xanthine oxidase. Shown is one subunit of the homodimeric protein, with the molybdenum-binding portion of the protein in gray, the domain containing the two iron-sulfur clusters in red and the domain containing the FAD in yellow. It is evident that the iron-sulfur clusters intervene between the molybdenum center and FAD. After Pai, Nishino and coworkers [55] (PDB 1FIQ).

In the time between our ESEEM and ENDOR work, protein crystal structures (finally) began to appear. First, the structure of the molybdenum-containing aldehyde oxidoreductase from *Desulfovibrio gigas* was determined by Robert Huber and Maria João Romão at the Max Planck Institute in Martinsried, working with José and Isabel Moura at the New University of Lisbon [54], followed several years later by the structure of milk xanthine oxidase itself by Emil Pai at the University of Toronto and Takeshi Nishino at Nippon Medical School [55]. I remember thinking “Yeah, right” when Takeshi told me the trick that had finally allowed crystallization was to use enzyme purified from the milk of a single cow. As it turns out, he was exactly right. On examining the crystal structure (Figure 9), it was extremely gratifying to see that the iron-sulfur clusters did indeed intervene physically between the molybdenum center and FAD, as our earlier pulse radiolysis work had predicted. In addition to establishing the overall folds of the polypeptide domains containing the several redox-active centers, the structure demonstrated that the molybdenum center of oxidized enzyme had a square-pyramidal coordination geometry, with a terminal Mo=X ligand in the apical position, and four additional ligands in the xy plane: two sulfurs from a pyranopterin cofactor, a Mo-OH and an equatorial Mo=Y ligand. Although the apical ligand was originally assigned to be the catalytically required Mo=S previously identified by XAS and the equatorial ligand a Mo=O, this assignment was questioned by inorganic chemists such as Martin Kirk at the University of New Mexico, who argued that the more stable configuration would have the stronger Mo=O ligand in the apical position. It is now generally accepted that it is indeed the Mo=O that occupies the apical position. The structure of the pyranopterin cofactor, which was recognized to be common to all molybdenum- and tungsten-containing enzymes (other than nitrogenase), had previously been established with the structure of the tungsten-containing aldehyde oxidoreductase from *D. gigas* by Michael Chan and Doug Rees at CalTech, working with Michael Adams and coworkers at the University of Georgia [56]. This cofactor, first inferred from the existence of nitrate reductase deficient nit-1 mutants of *Neurospora crassa* [57] and later extensively characterized by K.V. Rajagopalan at Duke University [58,59], consisted of the two-membered pterin ring elaborated by a third pyran ring containing a phosphate sidechain and a dithiolene moiety that coordinated to the metal in a bidentate fashion.

## 8. Bath, Brighton and Beyond

In light of this progress, Bob Bray had successfully arranged for a session on molybdenum enzymes at the 1997 annual meeting of the Biochemical Society, in Bath, England and I considered myself fortunate to have been invited to speak. It was an excellent scientific session, with contributions from Massey, Bray, Rajagopalan, Huber, Lowe and Nishino, among others. Bray had also arranged for a satellite meeting afterwards at the University of Sussex, near Brighton in the south of England, and had hired a bus to take all the participants at the Bath meeting. We stopped along the way to visit Stonehenge where we had the site essentially to ourselves for a couple of hours—an unforgettable experience. Bob had asked me to co-host the Sussex meeting, and in going over the list of invited speakers and other attendees I was extremely impressed at the number of European molecular biologists and microbiologists that were working on one or another aspect of molybdenum enzymes and molybdenum biology more generally. It was at this meeting that I first met, for example, Ralf Mendel and Rolf Thauer. I will always remember the atmosphere of excitement at both the scientific sessions and the social events of this meeting (the latter well-lubricated by a fine English bitter provided by a local brewery that Bray was in good with—I learned later he had a standing order for a keg to be dropped off at his home, which really impressed me). The one downside to the meeting was that it had unavoidably conflicted with an annual meeting of the American Chemical Society, and none of the US chemists active in the field at the time (e.g., John Enemark at Arizona, Jack Spence at Utah, Dick Holm at Harvard, Ed Stiefel at the Exxon Corporate Research Center) were able to attend. Stiefel in particular was excited about what he had heard of the meeting, and suggested that he and I submit an application to the Gordon Research Center to create a Gordon Conference focusing on molybdenum and tungsten enzymes. The application was successful, and the resulting Gordon Conferences ran in alternate years from 1999 to 2009. Since that time, it has been organized independently of the GRC, the most recent meeting being hosted by Partha Basu at Indiana University/Purdue University at Indianapolis in 2022. One aspect of these meetings that I am particularly proud of is that it (approximately) alternates between a European and North American venue, so that graduate students and post-docs working in the field on both continents are likely to have a chance to attend at least one meeting in the course of their studies.

## 9. Substrate Orientation and the Origin of Catalytic Power

The overall mechanism of substrate hydroxylation was by now well-established and generally accepted by the community. A new issue had arisen, however, as to the specific orientation of substrate in the active site. This was in fact a substantive matter as it had to do with the manner in which specific, highly conserved amino acid residues in the active site brought about the transition state stabilization that led to the observed rate acceleration seen in the enzyme-catalyzed reaction. These residues included: (1) a pair of phenylalanines between which substrate was sandwiched in the active site; (2) a glutamate that sat below the molybdenum center and was believed to function as an active site base, deprotonating the Mo-OH group to make it a better nucleophile in attacking the C-8 of substrate; (3) a second glutamate that sat above the phenylalanine residues in the substrate binding site; and (4) an arginine residue that sat at the bottom rear of the substrate binding site, on the opposite side of substrate from the molybdenum center.

**Figure 10:**
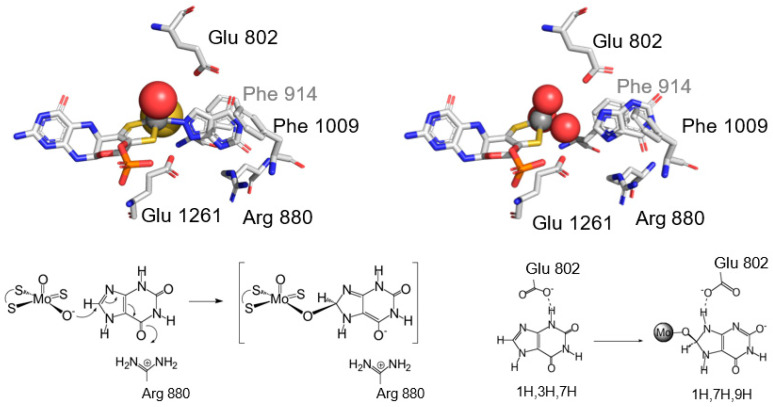
Alternate orientations of substrate in the active site of xanthine oxidase. **Upper left**, the “upside down” orientation seen with alloxanthine-complexed enzyme (compare with the orientation seen with the poor substrate 2-hydroxy-6-methylpurine in Figure 4). After Pai, Nishino and coworkers [60] (PDB 3BDJ). **Upper right**, the “right-side up” orientation seen with the good substrate xanthine bound to inactive, desulfo enzyme. After [61] (PDB 3EUB). **Bottom left**, stabilization of negative charge accumulation on substrate in the course of reaction by Arg 880. **Bottom right**, tautomerization of substrate in the course of the reaction as facilitated by Glu 802.

In our published mechanisms, we had drawn the substrate in what I will call a “right side up” orientation relative to the molybdenum center, and by inference the overall protein. This was in fact totally arbitrary on my part and the orientation was simply the way I had always drawn purines as a way to remember their structures. Working with Silke Leimkühler at the University of Potsdam, Germany and Takeshi Nishino, then at Nippon Medical School, we examined mutations at the two glutamates in the homologous xanthine dehydrogenase from *Rhodobacter capsulatus*. Mutation of Glu 730 (equivalent to Glu 1261 in the bovine enzyme) to any of several other residues reduced the limiting rate of reduction of enzyme at high [xanthine] from 67 s^−1^ to a rate that was undetectably slow, an effect of (minimally) 10^7^ in rate reduction or ~10 kcal/mol in compromised transition state stabilization, consistent with it functioning as a general base to initiate catalysis. The effect of mutating Glu 232 (corresponding to Glu 802 in the bovine enzyme) was less dramatic, reducing the limiting k_red_ by approximately an order of magnitude and increasing K_d_ by the same extent, indicating that this residue contributed ~1.5 kcal/mol in transition state stabilization and a comparable amount to substrate binding. Later work with Silke, again with the *R. capsulatus* enzyme, we demonstrated that mutating Arg 310 (equivalent to Arg 880 in the bovine enzyme) to methionine reduced k_red_ by ~10^4^, reflecting approximately 5.5 kcal/mol in compromised transition state stabilization; the effect of the R310K mutation was considerably less dramatic, with only a 20-fold reduction in k_red_. Interestingly, in comparing the effect of mutating Arg 310 on reactivity toward six different purines, the substrates were found to fall into two distinct groups, a first (including xanthine) that consisted of effective substrates with wild-type enzyme that were significantly affected by the R310M mutation, and a second that included much less effective substrates with wild-type enzyme but which were also much less affected by the mutation. We suggested that the good substrates bound in a “right side up” orientation in the active site so that Arg_310/880_ could stabilize negative charge accumulation on the C_6_=O oxygen in the course of the nucleophilic attack at C-8. The poor substrates bound in an “upside down” orientation that did not similarly stabilize charge accumulation, but in not being able to utilize Arg_310/880_, they were much less sensitive to its mutation. These orientations are shown in Figure 10.

In the meantime, Takeshi Nishino had proposed a mechanism that was essentially identical to ours but with the xanthine drawn inverted relative to our orientation, i.e., “upside down”. His main point was that both the tight-binding inhibitor alloxanthine and the slow substrate 2-hydroxy-6-methylpurine had in fact been shown crystallographically to bind in the “upside down” orientation. Alloxanthine binds rather differently than xanthine, however, coordinating directly to the molybdenum center via one of its pyrazole nitrogens (without an intervening oxygen) and lying more than 2 Å closer to the molybdenum than xanthine. Backing it out sufficiently to accommodate an intervening oxygen would require it to flip over to maintain the favorable interactions with Arg 880 and Glu 802 that are seen in the crystal structure [60]. As far as 2-hydroxy-methylpurine is concerned, it is after all an extremely slow substrate that is not nearly as rapidly hydroxylated by enzyme as is xanthine and its hydroxylation involves a substantially higher activation barrier, plausibly due to a less favorable binding orientation. Now, Takeshi and I went a long way back. He had also been a post-doctoral scholar with Vincent Massey a few years before me and had said more than once that we were family as a result. (I used to tell people this, then say that you didn’t notice the resemblance until Takeshi and I were standing next to one another.) We had a great many discussions about substrate orientation, some of which occurred in the course of a two-year sabbatical he spent with me at UC Riverside after his having retired from Nippon Medical School and while a new appointment for him was finalized at the University of Tokyo. The issue was settled, at least to my own satisfaction, when Hongnan Cao and James Pauff in my lab determined the structure of the inactive desulfo form of the enzyme with xanthine, where the substrate was found bound in our original “right side up” orientation, as shown in Figure 10. It is also shown how, with substrate bound in this way, Arg 880 is positioned so as to stabilize negative charge accumulation on substrate in the course of the initial nucleophilic attack, and Glu 802 positioned to facilitate tautomerization between N9 and N3, which would also contribute to transition state stabilization. Consistent with this, methylation at N3 essentially abolishes the ability of the enzyme to reduce 3-methylxanthine.

## 10. New Frontiers

At this point, the reaction mechanism of xanthine oxidase is considered well established (as the National Institutes of Health repeatedly reminded me in reviews of several unsuccessful grant renewals). Nevertheless, one question that remains outstanding with regard to xanthine oxidase that I would like to see answered is the manner in which the deeply buried molybdenum center is inserted into apoenzyme. Some have argued that insertion is through the 15 Å-long substrate access channel to the active site, and I recall watching a molecular dynamics simulation of cofactor binding showing that the cofactor was apparently able to negotiate the rather restricted channel to the substrate binding site, the pyranopterin moiety eventually becoming wedged between the two phenylalanine residues of the substrate binding site. The simulation stopped there, but the comment was made that the cofactor had made it to within 2 Å of its final position and was likely able to make it the rest of the way. It is true that the extracyclic amino group of the pyranopterin lay only ~2 Å from the position of the Mo-OH of holoenzyme at the end of the molecular dynamics simulation, but in the holoenzyme, the pyranopterin lies opposite the substrate binding site from the molybdenum—in fact, no atom of the cofactor was within ~15 Å of its final position in the holoenzyme at the end of the simulation. The molybdenum phosphate side chain of the pterin and the molybdenum complex itself had not been able to move past the pair of phenylalanines in the substrate binding site, and seemed sterically unable to do so. To my mind, the molecular dynamics simulation, far from proving that the cofactor could be inserted through the substate binding channel, demonstrated that it could not.

I have suggested an alternative, admittedly speculative, mechanism wherein the molybdenum cofactor is not inserted pterin-first through the substrate access channel, but instead molybdenum-first through the back of the protein [62]. This would require a substantial opening of the interface between the two domains that together bind the molybdenum center, an admittedly substantial change in protein configuration but one which might be triggered by binding of the sulfurase enzyme that is responsible for inserting the terminal sulfido group into the molybdenum coordination sphere [63]. This sulfuration is known to be the final step of cofactor maturation, and the sulfurase catalyzing the reaction has been implicated as being directly involved in insertion of the now mature cofactor into the apoenzyme as well. Insertion in this way might also provide an explanation as to why bacterial expression systems are ineffective in inserting the molybdenum center into eukaryotic proteins, since the region that the sulfurase would likely interact with the eukaryotic apoprotein on the “backside” of the molybdenum-binding portion of the protein has two inserts on the protein surface of 10–12 amino acid residues that are absent in the prokaryotic enzyme. These would plausibly interfere with cofactor insertion by the bacterial sulfurase into the eukaryotic apoenzyme. It remains to be seen whether there is any validity to the proposal. 

I have since happily moved on to other molybdenum-containing systems, including CO dehydrogenase and formate dehydrogenase. Reaction mechanisms we have proposed for each of these systems have so far stood the test of time, although with both these enzymes substantive questions remain (as to how, for example, specific active site residues contribute to transition state stabilization and rate acceleration). Most recently, we have taken up mechanistic studies of bifurcating flavoproteins. These fascinating and evolutionarily ancient systems take a pair of median-potential reducing equivalents from a source such as NADH and send each electron separately along high- and low-potential pathways in a way that is, overall, thermodynamically favorable. It is in this way that methanogenic archaea, acetogenic bacteria and other organisms generate the low-potential reducing equivalents to reduce ferredoxin for carbon and nitrogen fixation, as well as a variety of other cellular processes. It is still early days, but this work promises to be a productive line of enquiry for the indefinite future.

## Data Availability

No new data were created for this article.
